# Of mice and monkeys: using non-human primate models to bridge mouse- and human-based investigations of autism spectrum disorders

**DOI:** 10.1186/1866-1955-4-21

**Published:** 2012-07-30

**Authors:** Karli K Watson, Michael L Platt

**Affiliations:** 1 Department of Neurobiology, Duke University Medical Center, Durham, NC, 27710, USA; 2 Duke Institute for Brain Sciences, Duke University, Box 90999, Durham, NC, 27708, USA

**Keywords:** Autism, Asperger’s, Non-human primate, Monkey

## Abstract

The autism spectrum disorders (ASDs) arise from a diverse array of genetic and environmental origins that disrupt the typical developmental trajectory of neural connectivity and synaptogenesis. ASDs are marked by dysfunctional social behavior and cognition, among other deficits. Greater understanding of the biological substrates of typical social behavior in animal models will further our understanding of the etiology of ASDs. Despite the precision and tractability of molecular genetics models of ASDs in rodents, these organisms lack the complexity of human social behavior, thus limiting their impact on understanding ASDs to basic mechanisms. Non-human primates (NHPs) provide an attractive, complementary model for ASDs, due in part to the complexity and dynamics of social structures, reliance on vision for social signaling, and deep homology in brain circuitry mediating social behavior and reward. This knowledge is based on a rich literature, compiled over 50 years of observing primate behavior in the wild, which, in the case of rhesus macaques, is complemented by a large body of research characterizing neuronal activity during cognitive behavior. Several recent developments in this field are directly relevant to ASDs, including how the brain represents the perceptual features of social stimuli, how social information influences attention processes in the brain, and how the value of social interaction is computed. Because the symptoms of ASDs may represent extreme manifestations of traits that vary in intensity within the general population, we will additionally discuss ways in which nonhuman primates also show variation in social behavior and reward sensitivity. In cases where variation in species-typical behavior is analogous to similar variations in human behavior, we believe that study of the neural circuitry underlying this variation will provide important insights into the systems-level mechanisms contributing to ASD pathology.

## Review

### Introduction

**Figure 1:**
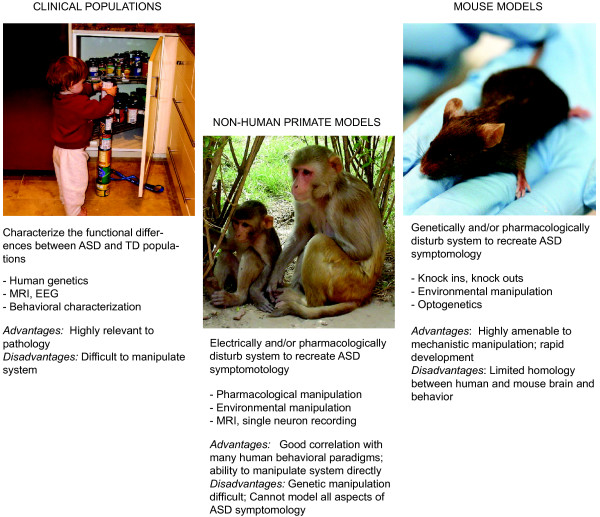
**A three-pronged approach to understanding and treating ASD.** Progress in any individual research domain (human, mouse, or primate-based studies) can be used to inform research directions in the other two domains. All images downloaded from Wikimedia Commons.

The autism spectrum disorders (ASDs) are behavioral syndromes characterized by communication deficits, repetitive behaviors, and altered social behavior [1]. Etiologically, ASDs are mysterious. Determining the cause of any ASD will require synthesis across several different models, encompassing both human and animal research. Each model offers its own set of advantages and disadvantages, but together they provide complementary and mutually informative sets of information. Studies of human clinical populations that directly test the behavioral, functional, and genetic characteristics correlated with ASD are a crucial part of the solution (Figure 1). Behavioral characteristics provide clues to the kinds of functional disruptions that cause the disorder, and whole-brain neural signatures provided by anatomical and functional MRI and EEG offer hints about which nodes of brain circuitry are the most heavily implicated in the disorders. However, the human model allows for few methods by which to manipulate the system in order to test causality, and even fewer methods that allow exploration of the molecular or cellular mechanisms of the disorder.

On the other end of the spectrum are mouse models, in which the symptoms of ASDs are re-created through the selective manipulation of genes, molecules, cells, or neural circuits (Figure 1). These two models inform one another: the identification of candidate genes, transmitter systems, or brain regions in human-based studies allows specific mechanisms to be systemically targeted in mouse models to test whether they result in ASD-like behaviors. Mouse models are advantageous in many respects, primarily because they are genetically tractable and appropriate for invasive studies. Knockout mouse variants, in which existing genes have been inactivated via genetic engineering, offer invaluable opportunities to test the functional and behavioral repercussions of manipulating a particular aspect of the nervous system. Other advantages of the mouse model include the features that make them suitable for “high-throughput” applications, such as brain slice in-vitro preparations. These features, including short gestation times, multiple births, and short life spans, also allow longitudinal or developmental studies to be completed within a short time frame.

### Limitations of mouse models

An ideal animal model of autism would be valid in three different domains. First, it would exhibit face validity, in which the behavior of the model is compromised in a manner consistent with ASD; second, etiological validity, that is, similarity to the underlying causes of the disorder; and, third, predictive validity, in which interventions effective in treating ASD induce the expected response in the model [2]. The latter two are nearly impossible to address, as the underlying cause of autism is unknown and there are no effective pharmacological treatments for the disorder, and even face validity can be difficult to establish in rodent models [3]. The onus is on the experimenter to determine which species-typical behaviors are analogous (or homologous) to those interrupted in autism and to demonstrate that experimentally induced behavioral impairment can be plausibly linked to autistic traits. This is no easy task, especially because many of the behaviors interrupted in ASD, such as language difficulties, are human-specific. Silverman and colleagues [4] review the types of behavioral assays used in conjunction with knockout mice that have provided clues regarding the molecular and cellular substrates underlying ASD. These include assays of (1) stereotypic behavior and resistance to change, including measures of repetitive self grooming and digging, displays of repetitive circling behavior, and perseveration; (2) social behavior, such as measures of social approach and preference, nose-to-nose sniffing, and social transmission of food preference; and (3) social communication, such as altered scent marking and ultrasonic vocalization patterns.

Given the expansion of the social behavior repertoire and concomitant elaboration of neural circuitry in primates (see below), it should not be assumed that rodent models of autism involving social behavioral phenotypes necessarily have high face validity. The behavioral assays described above are a valuable first step in validating an animal model of autism, especially when any single rodent model simultaneously presents altered behavior in several of them. However, the assays are crude, and their outcomes can be difficult to interpret. For example, decreased nose-to-nose sniffing could result from greater global anxiety, decreased social interest, or even deficits in olfactory perception. An increase in aggression could result from behavioral disinhibition, decreased ability to discriminate social cues, or an increased sensitivity to cues that elicit aggression. Lower rates of ultrasonic vocalization in mouse pups could be interpreted as a decreased tendency to communicate socially, or they could be indicative of lower anxiety. Assays of behavioral inflexibility and repetitive behavior often merit similar concerns. For example, one metric of behavioral inflexibility involves reversal-learning performance in a t-maze, in which, after learning which arm of the maze is baited with a reward, the reward is then switched to the other arm and the mouse must reverse its reward-seeking behavior accordingly [2,5,6]. It is arguable whether this assay is reflective of inflexible behavior in ASD, especially since behavioral findings of cognitive inflexibility on analogous tasks in ASD have been highly inconsistent [7].

One relatively under-used animal model that would help bridge the gap between the human and mouse-model-based approaches described above is the non-human primate (NHP; Figure 1). Because of their high degree of correspondence to human behavior, the outcomes of NHP behavioral assays are more readily interpreted than their rodent counterparts. In particular, NHPs have reasonable behavioral correlates to the human behaviors disrupted in autism, such as repetitive behaviors [8,9], social communication [10-13], and directed visual attention to the face and eyes [14]. The rich history of research on primate social behavior [11,12] provides great insight into the similarities and differences between human and monkey social behavior, and suggests that the similarities can be harnessed in the laboratory to develop behavioral tasks that are simultaneously relevant to behavioral disruptions induced by ASD and ethologically relevant to monkeys. The ability to manipulate neural mechanisms from the “bottom-up” in monkeys is subject to more constraints than mouse models, but is greatly widened in comparison to studies in human populations. For example, genetic knockouts do not exist in primates, but pharmacological approaches, environmental manipulations, and neural circuit study on the single cell level provide rich opportunities to inform and refine the mouse and human research. In the remainder of this review, we offer some of the ways in which NHP assays, in conjunction with pharmacologic or system-level manipulations (e.g., stimulation), could be used to advance the current state of knowledge about the etiology of ASD and to explore treatment development. For example, administrations of brain-site-specific oxytocin (OT) agonists or antagonists, serotonin system manipulations, or agents altering brain excitability are three ways in which the mechanistic findings from mouse model systems could be refined in nonhuman primates.

### Non-human primate models of ASD

A diminished capacity for social responsivity is probably the most disturbing aspect of ASD [15]. In order to understand how the “social brain” is affected in ASD, it is necessary to have a basic understanding of how these neural substrates operate in healthy individuals. The prefrontal cortices, amygdala, and temporoparietal regions contribute to social behavior in humans [16]. Prefrontal cortex, including anterior cingulate cortex, is involved in selecting the appropriate behavior based on its anticipated value, and is activated during tasks involving mentalizing and self knowledge [17]; the amygdala contributes to tagging emotionally relevant objects in the environment; and the temporo-parietal regions play a role in perceiving [18] and orienting towards visually salient information.

Humans and nonhuman primates show striking homology in the anatomy of neural circuits mediating social behavior. For example, while human prefrontal regions contain both granular and agranular cortex, allowing orbitofrontal cortex, anterior cingulate cortex, and dorsolateral cortex to be distinguished from one another, rat frontal cortex is exclusively agranular, making these distinctions impossible [19]. In fact, some researchers use these architectonic differences as evidence that rats and mice lack some prefrontal subregions found in primates altogether, such as dorsolateral prefrontal cortex [20] (but see [21]). If true, this is a serious concern, given the role of prefrontal cortex in social processing and its potential dysfunction in ASD [22]. Monkeys, by contrast, possess both dysgranular and agranular prefrontal cortex, and the major areas identified in humans by Brodmann are all identifiable in monkeys as well [20]. Other differences also exist; for example, astroglia with intralaminar processes are present in primate, but not rodent, brains [23].

Unlike rodents, both human and non-human primates rely primarily on visual cues in order to extract information from their social environments. This similarity allows similar paradigms to be used in both humans and monkeys, which will be useful for translation of therapeutics. For example, an ethologically relevant measure of pro-sociality in mice might consist of social sniffing displays, for which there is no obvious analog in humans. In contrast, in both monkeys [14], and in humans with ASD [24], the amount of visual attention to the eyes of another is a reliable metric of social behavior, and both are affected by intranasal applications of the neuropeptide oxytocin (OT) [25,26]. Neural manipulations in non-human primates that affect behavior in a manner reminiscent of ASD can thus be used as either a tool to study the etiology of ASD, or as a model for testing therapeutic agents that ameliorate ASD symptoms.

A third advantage to the use of nonhuman primates as models for understanding autism is that they have a large behavioral repertoire with a high degree of isomorphism to human behavior [27]. This is particularly true in the case of social behavior, and despite some differences (such as absence of biparental care and monogamous mating in macaques), the social ecologies of humans and rhesus macaques are quite similar. In the wild, rhesus macaques aggregate into large (~30-150 member), hierarchically organized social groups. Within these groups, dominance status and the extent of social integration determine each individual’s access to scarce resources such as food, water, and high-quality mates. Accordingly, rhesus macaques are socially savvy [28] and rely heavily on their ability to rapidly and accurately assess social situations and produce appropriate behavior. Both macaques and humans use visual cues to assess reproductive quality [29,30], regulate behavior according to the dominance rank of other individuals [31-33], and discriminate between in-group and out-group members [34,35].

These observations suggest monkeys evaluate other individuals in the environment and use this information to select the most advantageous behavior. Thus, social information appears to have intrinsic value to primates, demonstrated by the fact that both rhesus macaques [36,37] and humans [38] will work to view visual information about others. The relative value assigned to various classes of social information can also be measured in the laboratory in both humans and macaques. For example, male and female rhesus macaques systematically and spontaneously value visual social information, such as images of high-ranking male faces and the sexual skin of opposite sex conspecifics [31,39]. In humans, orbitofrontal cortex (OFC), ventromedial prefrontal cortex (vmPFC), and ventral striatum (VS) contribute to the computation of social value from images [40]. Attention-related neurons in parietal cortex signal the value of orienting to specific social stimuli in macaques as well [41]. Together, such studies sketch a neural circuit from input (temporal cortex) to value computation (OFC, vmPFC, VS), to output (parietal cortex) [42].

**Figure 2:**
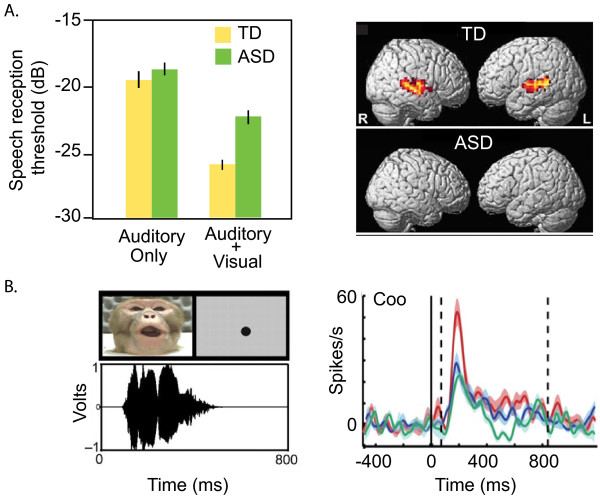
**Integration of visual and auditory information is commonplace in both humans and rhesus macaques, and is deficient in individuals with ASD.** (**A**) Behavioral and fMRI studies reveal differences in multisensory integration in ASD. Left, ASD and TD individuals perform similarly when discriminating speech sounds using auditory information alone, but ASD individuals are significantly impaired relative to TD individuals when visual information is added to the task. Speech information consisted of short sentences read aloud overlaid on a background of auditory noise. Y-axis, speech reception threshold, the speech-to-noise ratio at which individuals accurately report the speech signal. More negative values indicate better performance. Right, activity in the STS during audiovisual integration of speech is absent in ASD subjects. Images modified from [46,47]. (**B**) Single neurons of rhesus macaques represent audio-visual integration while perceiving meaningful vocalizations. Left, image and corresponding spectrogram of rhesus macaque performing a coo vocalization. Black dot on gray background is a visual control stimulus. Right, firing of a single STS neuron in response to hearing a coo (green), observing a coo (blue), or simultaneously hearing and observing a coo (red). Y-axis indicates the firing frequency of the neuron (spikes/second); X-axis indicates time, with coo stimulus presented at time zero. Note that higher neuronal firing is elicited when auditory and visual information is presented simultaneously. Images reproduced from [44].

Despite being highly visual, non-human primates communicate a great deal of information though vocalizations, including identity, sex, status, and reproductive quality [10]. To our knowledge, this sophisticated vocal communication in macaques has not been exploited as a model of social processing deficits in ASD, despite obvious relevance to verbal and non-verbal communication in humans. Moreover, recent studies in nonhuman primates have made progress in understanding how multimodal social information is processed in the primate brain. Given deficits in multisensory integration that occur in ASD [43], such research is highly relevant to understanding ASD. Neurons in the monkey auditory cortex and superior temporal sulcus region bind together auditory and visual information in order to provide an integrated representation of social communication [44]. For example, neurons in the superior temporal sulcus (STS) that respond to a specific type of species-typical vocalization, a coo, display enhanced firing in the presence of a movie depicting a monkey emitting the vocalization (Figure 2). Notably, STS function is altered in ASD individuals responding to visual social cues [45].

Mirror neurons, motor neurons that discharge when a subject both performs a motor act and observes another agent perform the same act, were first described in rhesus macaques [48]. The existence of these neurons in humans is inferred from fMRI studies showing that brain regions in which mirror neurons have been found in monkeys, such as inferior frontal cortex and rostral parietal cortex, are active in humans when both performing and observing a motor act [48]. The mirror neuron system (MNS) has been proposed to be dysfunctional in individuals with ASD (Iacoboni and Dapretto 2006). Though recent evidence casts doubt on this theory in the strict motor sense [49], individuals with ASD do have decreased activity in MNS relative to controls when imitating and observing emotional expressions, and the amount of BOLD suppression is correlated with the degree of social impairment [50].

**Figure 3:**
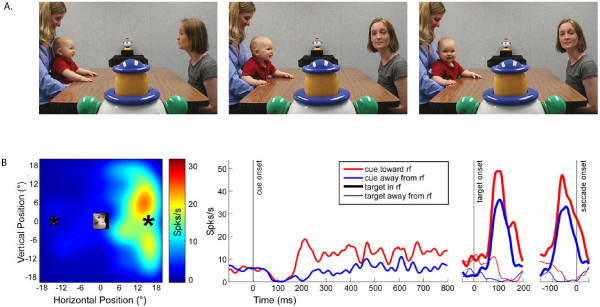
**Both humans and monkeys follow others’ gazes, a tendency that is reduced in autism.A**. Gaze-following, which occurs as early as 3 months of age in humans, promotes the phenomenon of joint visual attention. Image from [59]**B**. Social gaze enhances neural firing in lateral intraparietal cortex (LIP) during a visual target selection task. Left, LIP neurons in rhesus macaques are sensitive to particular locations in space. Here, the location of one of these so-called “response fields” is depicted for a single LIP neuron. Firing frequencies (hotter colors = higher firing rates, cooler colors = lower firing rates, in spikes per second) are overlaid in the form of a colorimetric map onto the visual scene. This particular neuron fires most when the monkey makes an eye movement to the right part of the monitor. Right, peri-stimulus time histogram of the same neuron firing when the eye movement is preceded by a picture of a monkey looking towards the response field (thick red line) or away from the response field (thick blue line). X-axis denotes time during a single trial, aligned at zero to cue, target, or saccade (eye movement) onset. Y-axis is spikes per second, i.e., the mean firing rate for this neuron. Note the increase in neuronal firing in response to an image of a familiar monkey looking towards the response field. Similar to humans, rhesus macaques exhibit gaze-following tendencies, as evidenced by decreased response times when monkeys saccade towards a target accompanied by a congruent social gaze stimulus. Image reproduced from [51].

It is possible that the mirror neuron motor system is a specific case of a more general mechanism that evolved to support other-oriented behavior in primates. For example, neurons in the lateral intraparietal (LIP) region of the macaque respond preceding gaze shifts to a particular region of space. Recently, investigators found that these neurons also respond when monkeys observe another monkey shift gaze to the same region [51] (Figure 3). Behaviorally, facilitation of gaze orientation via social cues is well known in humans: when we see a group of people swivel their heads to attend to something outside of our view, our natural tendency is to shift our gaze in the same direction in order to see what they are looking at. In typically developing (TD) individuals, observation of another’s gaze shift tends to induce re-orientation to the same region in space [52-56]. In ASD individuals, however, this tendency is often found to be altered [57] or impaired, e.g., [58].

Gaze-following is a precursor to joint attention, the simultaneous engagement of two or more people in the same object or event. Joint attention is reliably present at 18 months of age in typically developing individuals [60], but is one of the deficits in social interaction typical of ASD. The degree to which infants engage in joint attention can, to some extent, predict the probability of developing ASD later in development [61]. In TD individuals, gaze following occurs as early as 3-6 months old [60,62]. Because social attention tasks in human and nonhuman primates can be implemented in precisely the same way, the nonhuman primate model serves as an attractive tool for the identification of pharmacological interventions promoting social attention. Those interventions deemed effective (and safe) can then be easily tested in a human clinical population.

The molecular substrates mediating social affiliation in mammals appear to be relatively invariant and highly relevant to potential therapeutic treatments for ASD. Oxytocin (OT), a peptide hormone produced in the hypothalamus, has been implicated in ASD. For example, ASD has been linked to a mutation in the coding region for the OT receptor [63], and intranasal application of OT in ASD individuals increases attention to the eye region of faces [25], facilitates sensitivity to cooperative behavior [25], and improves emotional recognition [25,64]. Far from being specific to humans, OT is a primitive peptide that shapes social behavior in many other species [65], such as mother-offspring bonding in sheep [66] and monogamous pair bonds in prairie voles [67]. Oxytocin receptor knockout (OTR-KO) mice display social amnesia [68], impaired sociability, and reduced vocalization. The social deficits in OTR-KO mice are rescued by the administration of oxytocin [69]. Even in the (famously despotic) rhesus macaque, inhaled OT has the effect of increasing prosocial decisions in non-competitive contexts as well as attention to another individual [26].

fMRI studies reveal that the regions of the human brain affected by OT administration overlap heavily with those involved in social cognition, including the amygdala, prefrontal cortex, and temporo-parietal junction reviewed in [70]. As a complement, animal models can provide more specific answers about the way in which OT influences neural circuits related to social behavior. In humans, the gene encoding the OT receptor is polymorphic [71]. These and other polymorphisms may underlie some of the variation in human social behavior, and studies designed to explore this relationship may yield insights about the ways that OT can be used to treat ASD symptomology. Although we include a detailed discussion of OT research as it relates to ASDs, it is just one of many possible molecular mechanisms that could be further explored in NHPs. Serotonergic pathways [72] and excitation/inhibition balances in nervous systems [73] are examples of two additional mechanisms that, based on mouse-model evidence, may play a role in ASD pathology, and merit further exploration in NHP models.

### Repetitive behaviors

Although the bulk of the research on ASD focuses on social disruptions, it is important to note that non-social alterations in behavior, such as motor and verbal stereotypies, resistance to change, and obsessive interests, are equally characteristic of the disorder [1,74,75]. Indeed, circumscribed interests and repetitive behavior interfere greatly with normal function, and are a major source of stress amongst parents of ASD children [76]. Mouse assays that index levels of repetitive/stereotyped motor behavior include increased rates of self-grooming and bar-biting [4], and repetitive digging behavior as measured by marble-burying assays [77]. Resistance to change may be assayed by T-maze reversal learning and water maze tasks [2].

Interestingly, despite being known for its effects on social behavior (see discussion above), OT can also affect patterns of repetitive behavior. OTR-KO mice are resistant to change as measured by a T-maze reversal learning task, but, remarkably, cognitive flexibility is restored by OT administration [69]. The OTR-KO mouse model of autism thus comes very close to having predictive, as well as face and construct, validity, as OT administration is known to reduce repetitive behavior in humans with ASD [78].

Stereotyped behavior has also been described in captive primates, and measures of these behaviors are under-used but highly relevant to NHP models of ASD. In one of the rare studies to quantify repetitive behaviors in NHP models of ASD, monkeys exposed to human IgG antibodies collected from mothers with multiple children diagnosed with ASD showed increased whole-body stereotypies and were hyperactive compared to control monkeys [9]. When placed in an enclosure with visual access to their mother, control animals sat in close proximity to their mother, whereas IgG-exposed animals repeatedly paced the length of the enclosure. Moreover, the IgG-exposed animals displayed stereotyped body-flipping behavior, even in large enclosures that offered opportunities for play and exploration.

Captive rhesus macaques housed in isolation often exhibit behavioral stereotypies such as repeated pacing and flipping [8]. The effects of social deprivation in nonhuman primates mirror those seen in humans; neonates raised in conditions of privation and absence of maternal care often show autistic-like behavior [79]. These observations invite the speculation that repetitive behavior in ASD is a consequence of self-induced social isolation.

### Modeling neurodevelopmental disorders in nonhuman primates

In addition to the advantages delineated above, NHP models also provide unique advantages when exploring neurodevelopmental contributors to autism, such as disruption in brain growth and connectivity during development [80,81]. It seems likely that ASD arises from a gene-environment interaction, and the timing of the environmental insult may be crucial to development of ASD. In contrast to rodents, rhesus macaques bear single young with a lengthy period of dependence and postnatal maturation. Classic experiments by Harlow [82] demonstrated that social interaction is required for normal emotional development in macaques. Peer-reared rhesus macaques with amygdala lesions show social withdrawal and a decrease in initiation and acceptance of social contacts as adults [83]. Subsequent studies showed that amgydala lesioning alone was not sufficient to induce social dysfunction, and that maternally reared infants with amygdala lesions retained intact social gaze, facial expression, body posture, and social interest [84]. This outcome highlights the importance of interactions between the environment and functional risk factors to produce alterations in primate behavior.

Insults during prenatal development are also implicated in ASD. Studies in rhesus macaques and mice partially support an autoimmune model of autism driven by exposure to maternal antibodies in *utero*[85]. As in humans, rhesus macaques and other nonhuman primates transfer maternal immunoglobulins across the placenta during gestation, whereas rodents receive immunity postnatally [86]. The degree to which the mother and the fetus intermingle depends on the anatomy of the placenta, which varies across species; it is highest in humans, intermediate in rhesus macaques, and minimal in rodents [87,88].

### Individual variation and ASD

Just as social behavior varies across the typically developing population, so does behavior within the ASD population (hence the term “spectrum”). Moreover, unaffected family members of individuals with ASD often exhibit “broader phenotypes,” milder versions of ASD symptomology that do not substantially impact functioning. Like humans, monkeys display notable individual variation in social behavior. For example, rhesus macaques who carry a copy of the short allele in the serotonin transporter linked repeat polymorphism direct less attention to the eyes than others, or are less likely to look at a face than a non-face image [14]. Variations in the degree of social integration are also documented among macaques in the wild, and can partially be explained by genetic factors. For example, social network analysis confirms that patterns of grooming and aggressive behaviors can be partially explained by repeat polymorphisms associated within the serotonin system [29]. The presence of such endophenotypes in macaques offers another dimension along which the biology of ASD symptomatology can be explored.

However, to our knowledge, individual variation of ASD-like traits has not been explored in mice, though heavy inbreeding has resulted in the amplification of ASD-like traits in some strains of laboratory mice. There are 11 commonly used laboratory mouse strains descended from a single mouse species, *Mus musculus*. Within each strain, each mouse is nearly genetically identical. Between each strain, however, there is a high level of genetic diversity contained within diversity “hot spots” in the genome [89]. The genetic differences between these different strains are sufficient to induce behavioral differences in tasks modeled to probe core ASD symptoms in mice. For example, BTBR mice display low social approach, poor social learning, and heightened resistance to change [5], as well as impaired probabilistic reversal learning and increased marble-burying and grooming behavior [90]. BALB/c mice display reduced rates of some species-typical social behaviors, such as copulation and maternal behaviors, as well as heightened anxiety and increased aggression [91]. Because there are many genetic differences between strains, it is not clear how these differences arise. However, as the genomic differences between strains become better characterized, it may be possible to home in on the genes and pathways that underlie ASD-like behaviors in the affected strains.

### Ethical considerations

For reasons outlined in this review, experiments in non-human primates have the capacity to contribute unique information about the relationship between the nervous system and ASD. However, the same characteristics that make non-human primates valuable for ASD studies warrant careful consideration of the ethical implications of such research. The use of animals in research has a rich history, reviewed in [92], and has resulted in the current system, in which experimental and husbandry procedures involving nonhuman primates are strictly regulated. These regulations notwithstanding, the use of intelligent animals in any research program demands close scrutiny, and different viewpoints on non-human primate research exist. These viewpoints must be considered in the context of recent studies that show a rapid increase in the prevalence of autism [93], as well as the associated financial [94] and social, e.g., [95,96], repercussions. In the authors’ opinions, the scientific advancements and the impact on autism treatments to be potentially gained by experimental research on non-human primates outweigh ethical concerns of such research.

## Conclusions

The ASDs are common, costly, and socially devastating, placing a premium on therapeutic progress. The complexity of the disorder demands a multi-pronged approach. We argue that a tripartite approach, integrating clinical studies in humans, genetic manipulations in mice, and neural systems studies in non human primates, offers the most promise for understanding and, ultimately, treating ASD. Mice offer an ideal substrate for bottom-up studies, in which the precise biological consequences of various genetic disruptions can be identified. Studies in nonhuman primates offer a complimentary top-down approach, appropriate for identifying the neural circuits and patterning associated with the behaviors affected in ASD. Addressing the disorder on all three levels, in humans, primates, and rodents, offers the most hope for a translatable therapy for ASD.

## Abbreviations

ASDs, autism spectrum disorders; NHPs, non-human primates; OT, oxytocin; OFC, orbitofrontal cortex; vmPFC, ventro-medial prefrontal cortex; VS, ventral striatum; MNS, mirror neuron system; STS, superior temporal sulcus; LIP, lateral intraparietal; OTR-KO, oxytocin receptor knockout.

## Competing interests

All authors declare that they have no competing interests.

## Authors’ contributions

KW drafted the manuscript and prepared the figures. MLP edited the manuscript. All authors read and approved the final manuscript.

## References

[B1] American Psychiatric Association Diagnostic and statistical manual of mental disorders: DSM-IV 1994

[B2] Crawley JN Designing mouse behavioral tasks relevant to autistic-like behaviors Ment Retard Dev Disabil Res Rev 2004 10 248 258 15666335 10.1002/mrdd.20039

[B3] Nestler EJ Hyman SE Animal models of neuropsychiatric disorders Nat Neurosci 2010 13 1161 1169 20877280 10.1038/nn.2647 PMC3750731

[B4] Silverman JL Yang M Lord C Crawley JN Behavioural phenotyping assays for mouse models of autism Nat Rev Neurosci 2010 11 490 502 20559336 10.1038/nrn2851 PMC3087436

[B5] Moy SS Nadler JJ Young NB Perez A Holloway LP Barbaro RP Barbaro JR Wilson LM Threadgill DW Lauder JM Mouse behavioral tasks relevant to autism: Phenotypes of 10 inbred strains Behav Brain Res 2007 176 4 20 16971002 10.1016/j.bbr.2006.07.030 PMC1857288

[B6] Moy SS Nadler JJ Young NB Nonneman RJ Segall SK Andrade GM Crawley JN Magnuson TR Social approach and repetitive behavior in eleven inbred mouse strains Behav Brain Res 2008 191 118 129 18440079 10.1016/j.bbr.2008.03.015 PMC2441761

[B7] Geurts HM Corbett B Solomon M The paradox of cognitive flexibility in autism Trends Cog Sci 2009 13 74 82 10.1016/j.tics.2008.11.006 PMC5538880 19138551

[B8] Lutz C Well A Novak M Stereotypic and self-injurious behavior in rhesus macaques: A survey and retrospective analysis of environment and early experience Am J Primatol 2003 60 1 15 12766938 10.1002/ajp.10075

[B9] Martin LA Ashwood P Braunschweig D Cabanlit M Van de Water J Amaral DG Stereotypies and hyperactivity in rhesus monkeys exposed to IgG from mothers of children with autism Brain Behav Immun 2008 22 806 816 18262386 10.1016/j.bbi.2007.12.007 PMC3779644

[B10] Ghazanfar AA Santos LR Primate brains in the wild: the sensory bases for social interactions Nat Rev Neurosci 2004 5 603 616 15263891 10.1038/nrn1473

[B11] Smuts BB, Cheney DL, Seyfarth RM, Wrangham RW, Struhsaker TT Primate societies 1987 1987:xi, 578

[B12] Cheney DL Seyfarth RM Baboon Metaphysics: The Evolution of a Social Mind 2008 University of Chicago Press, Chicago

[B13] Platt ML, Ghazanfar AA Primate Neuroethology 2010 Oxford University Press, New York

[B14] Watson KK Ghodasra JH Platt ML Serotonin transporter genotype modulates social reward and punishment in rhesus macaques PLoS ONE 2009 4 e4156 19142220 10.1371/journal.pone.0004156 PMC2612746

[B15] Hoppes K Harris SL Perceptions of child attachment and maternal gratification in mothers of children with autism and Down syndrome J Clin Child Psychol 1990 19 365 370

[B16] Adolphs R The social brain: Neural basis of social knowledge Annual Review of Psychology. Volume 60. Palo Alto: Annual Reviews 2009 693 716 Annual Review of Psychology 10.1146/annurev.psych.60.110707.163514 PMC2588649 18771388

[B17] Amodio DM Frith CD Meeting of minds: the medial frontal cortex and social cognition Nat Rev Neurosci 2006 7 268 277 16552413 10.1038/nrn1884

[B18] Allison T Puce A McCarthy G Social perception from visual cues: role of the STS region Trends Cog Sci 2000 4 267 278 10.1016/s1364-6613(00)01501-1 10859571

[B19] Ongur D Price JL The organization of networks within the orbital and medial prefrontal cortex of rats, monkeys and humans Cereb Cortex 2000 10 206 219 10731217 10.1093/cercor/10.3.206

[B20] Preuss TM Do rats have prefrontal cortex? The Rose-Woolsey-Akert program reconsidered J Cogn Neurosci 1995 7 1 24 10.1162/jocn.1995.7.1.1 23961750

[B21] Uylings HBM Groenewegen HJ Kolb B Do rats have a prefrontal cortex? Behav Brain Res 2003 146 3 17 14643455 10.1016/j.bbr.2003.09.028

[B22] Sugranyes G Kyriakopoulos M Corrigall R Taylor E Frangou S Autism spectrum disorders and schizophrenia: Meta-analysis of the neural correlates of social cognition PLoS ONE 2011 6 13 10.1371/journal.pone.0025322 PMC3187762 21998649

[B23] Colombo JA Fuchs E Hartig W Marotte LR Puissant V Rodent-like and primate-like types of astroglial architecture in the adult cerebral cortex of mammals: a comparative study Brain Struct Funct 2000 201 111 120 10.1007/pl00008231 10672363

[B24] Baron-Cohen S Wheelwright S Jolliffe aT: Is there a" language of the eyes"? Evidence from normal adults, and adults with autism or Asperger syndrome Visual Cog 1997 4 311 331

[B25] Andari E Duhamel JR Zalla T Herbrecht E Leboyer M Sirigu A Promoting social behavior with oxytocin in high-functioning autism spectrum disorders Proc Nat Acad Sci 2010 107 4389 20160081 10.1073/pnas.0910249107 PMC2840168

[B26] Chang SWC Barter JW Ebitz RB Watson KK Platt ML Inhaled oxytocin amplifies both vicarious reinforcement and self reinforcement in rhesus macaques (Macaca mulatta) Proc Nat Acad Sci 2012 109 959 964 22215593 10.1073/pnas.1114621109 PMC3271866

[B27] Dantzer R Kelley KW Autistic children: a neuroimmune perspective Brain Behav Immun 2008 22 804 805 18420377 10.1016/j.bbi.2008.03.001

[B28] Maestripieri D Macachiavellian Intelligence: How Rhesus Macaques and Humans Have Conquered the World Chicago 2007 University of Chicago Press,

[B29] Brent LJ Heilbronner SR Horvath JE Gonzanelz-Martinez J Ruiz-Lambides AV Robinson A Skene JHP Platt ML Genetics of social network position in free-ranging rhesus macaques Neuroscience 2011 Abstracts 2011 Society for Neuroscience, Washington, D.C

[B30] Higham JP Hughes KD Brent LJN Dubuc C Engelhardt A Heistermann M Maestriperi D Santos LR Stevens M Familiarity affects the assessment of female facial signals of fertility by free-ranging male rhesus macaques Proc Royal Soc B 2011 278 3452 3458 10.1098/rspb.2011.0052 PMC3177625 21471112

[B31] Deaner RO Khera AV Platt ML Monkeys pay per view: adaptive valuation of social images by rhesus macaques Curr Biol 2005 15 543 548 15797023 10.1016/j.cub.2005.01.044

[B32] Sidanius J Pratto F Social dominance: An intergroup theory of social hierarchy and oppression 1999 Cambridge University Press, New York, NY

[B33] Van Noordwijk MA Van Schaik CP Career moves: transfer and rank challenge decisions by male long-tailed macaques Behaviour 2001 138 359 395

[B34] Harris LT Fiske ST Dehumanizing the lowest of the low Psych Sci 2006 17 847 10.1111/j.1467-9280.2006.01793.x 17100784

[B35] Mahajan N Martinez MA Gutierrez NL Diesendruck G Banaji MR Santos LR The evolution of intergroup bias: Perceptions and attitudes in rhesus macaques J Pers Soc Psychol 2011 100 387 405 21280966 10.1037/a0022459

[B36] Andrews M Rosenblum L Live-social-video reward maintains joystick task performance in bonnet macaques Percept Motor Skills 1993 77 755 8284149 10.2466/pms.1993.77.3.755

[B37] Brannon EM Andrews MW Rosenblum LA Effectiveness of video of conspecifics as a reward for socially housed bonnet macaques (Macaca radiata) Percept Motor Skills 2004 98 849 858 15209299 10.2466/pms.98.3.849-858

[B38] Hayden BY Parikh PC Deaner RO Platt ML Economic principles motivating social attention in humans Proc Biol Sci 2007 274 1751 1756 17490943 10.1098/rspb.2007.0368 PMC2493582

[B39] Watson KK Ghodasra J Furlong MA Platt ML Visual preferences for sex and status in female rhesus macaques Anim Cogn 2012 15 401 407 22160645 10.1007/s10071-011-0467-5 PMC3518424

[B40] Smith DV Hayden BY Truong TK Song AW Platt ML Huettel SA Distinct value signals in anterior and posterior ventromedial prefrontal cortex J Neurosci 2010 30 2490 2495 20164333 10.1523/JNEUROSCI.3319-09.2010 PMC2856318

[B41] Klein JT Deaner RO Platt ML Neural correlates of social target value in macaque parietal cortex Curr Biol 2008 18 419 424 18356054 10.1016/j.cub.2008.02.047 PMC2362498

[B42] Klein JT Shepherd SV Platt ML Social attention and the brain Curr Biol 2009 19 R958 R962 19889376 10.1016/j.cub.2009.08.010 PMC3387539

[B43] Iarocci G McDonald J Sensory integration and the perceptual experience of persons with autism J Autism Dev Disord 2006 36 77 90 16395537 10.1007/s10803-005-0044-3

[B44] Ghazanfar AA Chandrasekaran C Logothetis NK Interactions between the superior temporal sulcus and auditory cortex mediate dynamic face/voice integration in rhesus monkeys J Neurosci 2008 28 4457 4469 18434524 10.1523/JNEUROSCI.0541-08.2008 PMC2663804

[B45] Pelphrey KA Morris JP McCarthy G Neural basis of eye gaze processing deficits in autism Brain 2005 128 1038 15758039 10.1093/brain/awh404

[B46] Smith EG Bennetto L Audiovisual speech integration and lipreading in autism J Child Psychol Psych 2007 48 813 821 10.1111/j.1469-7610.2007.01766.x 17683453

[B47] Gervais H Belin P Boddaert N Leboyer M Coez A Sfaello I Barthelemy C Brunelle F Samson Y Zilbovicius M Abnormal cortical voice processing in autism Nat Neurosci 2004 7 801 802 15258587 10.1038/nn1291

[B48] Rizzolatti G Craighero L The mirror-neuron system. Annu Rev Neurosci 2004 27 169 192 10.1146/annurev.neuro.27.070203.144230 15217330

[B49] Dinstein I Thomas C Humphreys K Minshew N Behrmann M Heeger DJ Normal movement selectivity in autism Neuron 2010 66 461 469 20471358 10.1016/j.neuron.2010.03.034 PMC2872627

[B50] Dapretto M Davies MS Pfeifer JH Scott AA Sigman M Bookheimer SY Iacoboni M Understanding emotions in others: mirror neuron dysfunction in children with autism spectrum disorders Nat Neurosci 2005 9 28 30 16327784 10.1038/nn1611 PMC3713227

[B51] Shepherd SV Klein JT Deaner RO Platt ML Mirroring of attention by neurons in macaque parietal cortex Proc Nat Acad Sci 2009 106 9489 19470477 10.1073/pnas.0900419106 PMC2685741

[B52] Langton SRH Watt RJ Bruce V Do the eyes have it? Cues to the direction of social attention Trends Cogn Sci 2000 4 50 59 10652522 10.1016/s1364-6613(99)01436-9

[B53] Langton SRH Bruce V Reflexive visual orienting in response to the social attention of others Visual Cogn 1999 6 541 567

[B54] Driver J Davis G Ricciardelli P Kidd P Maxwell E Baron-Cohen S Gaze perception triggers reflexive visuospatial orienting Visual Cogn 1999 6 509 540

[B55] Ristic J Friesen CK Kingstone A Are eyes special? It depends on how you look at it Psychon Bull Rev 2002 9 507 513 12412890 10.3758/bf03196306

[B56] Friesen CK Kingstone A The eyes have it! Reflexive orienting is triggered by nonpredictive gaze Psychon Bull Rev 1998 5 490 495

[B57] Vlamings PHJM Stauder JEA Van Son IAM Mottron L Atypical visual orienting to gaze-and arrow-cues in adults with high functioning autism J Autism Dev Disord 2005 35 267 277 16119468 10.1007/s10803-005-3289-y

[B58] Leekam S Baron-Cohen S Perrett D Milders M Brown S Eye-direction detection: a dissociation between geometric and joint attention skills in autism Br J Dev Psychol 1997 15 77 95

[B59] Meltzoff AN Kuhl PK Movellan J Sejnowski TJ Foundations for a new science of learning Science 2009 325 284 19608908 10.1126/science.1175626 PMC2776823

[B60] Corkum V Moore C Moore C, Dunham PJ, Dunham P Development of joint visual attention in infants Joint attention: Its Origins and Role in Development 1995 Lawrence Erlbaum Associates, Inc, Hillsdale 61 83

[B61] Baird G Charman T Baron-Cohen S Cox A Swettenham J Wheelwright S Drew A A screening instrument for autism at 18 months of age: a 6-year follow-up study J Am Acad Child Adolesc Psych 2000 39 694 702 10.1097/00004583-200006000-00007 10846303

[B62] D'Entremont B Hains S Muir D A demonstration of gaze following in 3-to 6-month-olds Infant Beh Dev 1997 20 569 572

[B63] Wu S Jia M Ruan Y Liu J Guo Y Shuang M Gong X Zhang Y Yang X Zhang D Positive association of the oxytocin receptor gene (OXTR) with autism in the Chinese Han population Biol Psych 2005 58 74 77 10.1016/j.biopsych.2005.03.013 15992526

[B64] Guastella AJ Einfeld SL Gray KM Rinehart NJ Tonge BJ Lambert TJ Hickie IB Intranasal oxytocin improves emotion recognition for youth with autism spectrum disorders Biol Psych 2010 67 692 694 10.1016/j.biopsych.2009.09.020 19897177

[B65] Insel TR The challenge of translation in social neuroscience: a review of oxytocin, vasopressin, and affiliative behavior Neuron 2010 65 768 779 20346754 10.1016/j.neuron.2010.03.005 PMC2847497

[B66] Keverne EB Kendrick KM Oxytocin facilitation of maternal behavior in sheep Ann N Y Acad Sci 1992 652 83 101 1385685 10.1111/j.1749-6632.1992.tb34348.x

[B67] Insel TR Hulihan TJ A gender-specific mechanism for pair bonding: Oxytocin and partner preference formation in monogamous voles Behav Neurosci 1995 109 782 7576222 10.1037//0735-7044.109.4.782

[B68] Ferguson JN Young LJ Hearn EF Matzuk MM Insel TR Winslow JT Social amnesia in mice lacking the oxytocin gene Nat Genet 2000 25 284 288 10888874 10.1038/77040

[B69] Sala M Braida D Lentini D Busnelli M Bulgheroni E Capurro V Finardi A Donzelli A Pattini L Rubino T Pharmacologic rescue of impaired cognitive flexibility, social deficits, increased aggression, and seizure susceptibility in oxytocin receptor null mice: a neurobehavioral model of autism Biol Psych 2011 69 875 882 10.1016/j.biopsych.2010.12.022 21306704

[B70] Zink CF Meyer-Lindenberg A Human neuroimaging of oxytocin and vasopressin in social cognition Horm Behav 2012 61 400 409 22326707 10.1016/j.yhbeh.2012.01.016 PMC3312952

[B71] Michelini S Urbanek M Dean M Goldman D Polymorphism and genetic mapping of the human oxytocin receptor gene on chromosome 3 Am J Med Genetics 1995 60 183 187 7573168 10.1002/ajmg.1320600303

[B72] Veenstra-VanderWeele J Muller CL Iwamoto H Sauer JE Owens WA Shah CR Cohen J Mannangatti P Jessen T Thompson BJ Autism gene variant causes hyperserotonemia, serotonin receptor hypersensitivity, social impairment and repetitive behavior Proc Nat Acad Sci 2012 109 5469 5474 22431635 10.1073/pnas.1112345109 PMC3325657

[B73] Auerbach BD Osterweil EK Bear MF Mutations causing syndromic autism define an axis of synaptic pathophysiology Nature 2011 480 63 68 22113615 10.1038/nature10658 PMC3228874

[B74] Sasson NJ Elison JT Turner-Brown LM Dichter GS Bodfish JW Brief Report: Circumscribed attention in young children with autism J Autism Dev Dis 2011 41 242 247 10.1007/s10803-010-1038-3 PMC3709851 20499147

[B75] Lam KSL Bodfish JW Piven J Evidence for three subtypes of repetitive behavior in autism that differ in familiality and association with other symptoms J Child Psychol Psychiatry 2008 49 1193 1200 19017031 10.1111/j.1469-7610.2008.01944.x PMC3709850

[B76] Klin A Danovitch JH Merz AB Volkmar FR Circumscribed interests in higher functioning individuals with autism spectrum disorders: An exploratory study Res Pract Pers Sev Disabil 2007 32 89 100

[B77] Thomas A Burant A Bui N Graham D Yuva-Paylor LA Paylor R Marble burying reflects a repetitive and perseverative behavior more than novelty-induced anxiety Psychopharmacology 2009 204 361 373 19189082 10.1007/s00213-009-1466-y PMC2899706

[B78] Hollander E Novotny S Hanratty M Yaffe R DeCaria CM Aronowitz BR Mosovich S Oxytocin infusion reduces repetitive behaviors in adults with autistic and Asperger's disorders Neuropsychopharmacology 2003 28 193 198 12496956 10.1038/sj.npp.1300021

[B79] Rutter ML Kreppner JM O'Connor TG Specificity and heterogeneity in children's responses to profound institutional privation Br J Psych 2001 179 97 103 10.1192/bjp.179.2.97 11483469

[B80] Machado CJ Bachevalier J Non-human primate models of childhood psychopathology: the promise and the limitations J Child Psychol Psych 2003 44 64 87 10.1111/1469-7610.00103 12553413

[B81] Pardo CA Eberhart CG The neurobiology of autism Brain Pathol 2007 17 434 447 17919129 10.1111/j.1750-3639.2007.00102.x PMC8095519

[B82] Harlow HF The nature of love Am Psychol 1958 13 673 685

[B83] Bachevalier J Medial temporal lobe structures and autism: a review of clinical and experimental findings Neuropsychologia 1994 32 627 648 8084420 10.1016/0028-3932(94)90025-6

[B84] Amaral D Bauman M Mills Schumann C The amygdala and autism: implications from non-human primate studies Genes Brain Behav 2003 2 295 302 14606694 10.1034/j.1601-183x.2003.00043.x

[B85] Patterson PH Maternal infection: window on neuroimmune interactions in fetal brain development and mental illness Curr Opin Neurobiol 2002 12 115 118 11861174 10.1016/s0959-4388(02)00299-4

[B86] Pentšuk N van der Laan JW An interspecies comparison of placental antibody transfer: new insights into developmental toxicity testing of monoclonal antibodies Birth Defects Res B 2009 86 328 344 10.1002/bdrb.20201 19626656

[B87] Carter AM Animal models of human placentation: A review Placenta 2007 28 Supplement S41 S47 17196252 10.1016/j.placenta.2006.11.002

[B88] Ankel-Simons F Primate Anatomy: An Introduction 2007 3 Academic, Burlington

[B89] Yang H Bell TA Churchill GA de Villena FP-M On the subspecific origin of the laboratory mouse Nat Genet 2007 39 1100 1107 17660819 10.1038/ng2087

[B90] Amodeo DA Jones JH Sweeney JA Ragozzino ME Differences in BTBR T plus tf/J and C57BL/6 J mice on probabilistic reversal learning and stereotyped behaviors Behav Brain Res 2011 227 64 72 22056750 10.1016/j.bbr.2011.10.032 PMC3273330

[B91] Brodkin ES BALB/c mice: low sociability and other phenotypes that may be relevant to autism Behav Brain Res 2007 176 53 65 16890300 10.1016/j.bbr.2006.06.025

[B92] Baumans V Use of animals in experimental research: an ethical dilemma? Gene Ther 2004 11 S64 S66 15454959 10.1038/sj.gt.3302371

[B93] Baio J Prevelance of Autism Spectrum disorders — Autism and developmental disabilities monitoring network, 14 Sites, United States, 2008 CDC Morb Mortal Weekly Report 2012 61 1 19 22456193

[B94] Ganz ML The lifetime distribution of the incremental societal costs of autism Arch Pediatr Adolesc Med 2007 161 343 E345 17404130 10.1001/archpedi.161.4.343

[B95] Hare DJ Pratt C Burton M Bromley J Emerson E The health and social care needs of family carers supporting adults with autistic spectrum disorders Autism 2004 8 425 444 15556960 10.1177/1362361304047225

[B96] Wolf LC Noh S Fisman SN Speechley M Psychological effects of parenting stress on parents of autistic children J Autism Dev Disord 1989 19 157 166 2523379 10.1007/BF02212727

